# Round Cells in Diagnostic Semen Analysis: A Guide for Laboratories and Clinicians

**DOI:** 10.3389/bjbs.2021.10129

**Published:** 2022-02-02

**Authors:** S. Long, S. Kenworthy

**Affiliations:** ^1^ University Hospitals Birmingham, Birmingham, United Kingdom; ^2^ Retired, Portsmouth, United Kingdom

**Keywords:** infection, round cells, semen, sperm, WBC, analysis, germ cells

## Abstract

Round cells in seminal fluid are defined as either leucocytes or immature germ cells. Laboratories undertaking semen analysis often report these combined as a concentration, with no further review, comment or direction for clinician action or review. Although numerous publications discuss the possible clinical relevance of these cells (particularly leucocytes) in infertility, the methods employed to differentiate them are often beyond the scope of most diagnostic laboratories. This paper aims to support healthcare scientists in understanding the clinical significance of round cells and aid their identification, differentiation and interpretation. This will support the quality of care the patient receives and direct clinicians to further considerations that may be appropriate for their patient and should consequently reduce indiscriminate and unnecessary use of antibiotics.

## Introduction

The first World Health Organisation (WHO) *Laboratory manual for the examination of human semen and sperm-cervical mucus interaction* was introduced in 1980 (1), and the sixth edition, now entitled *WHO laboratory manual for the examination and processing of human semen*, (WHO 2021) was released in July 2021 (2). Whilst most laboratories will align diagnostic semen analysis (DSA) with current WHO guidelines, DSA techniques and clinical treatment have barely progressed compared with the advancement in research surrounding cellular and molecular sperm function (3).

WHO 2021 covers all aspects of semen analysis, alongside additional “optional” tests. The estimation of round cell concentration is included in some basic semen analysis procedures. Leucocytes and immature germ cells present in semen are collectively defined as “round cells” (2). Leucocytes, otherwise known as white blood cells, include granule-containing neutrophils, eosinophils and basophils, along with lymphocytes (T and B cells) and monocytes (precursors of macrophages), all which play a vital role in the body’s immune system (4). Germ cells are defined as the precursors of gametes, for example, spermatocytes in spermatogenesis (2). WHO 2021 details methods for round cell concentration estimation, and further specific techniques for the confirmation of these cell types. These include immunocytochemistry using peroxidase activity for leucocyte recognition (2). The manual acknowledges the difficulties in interpreting the clinical significance of the identification of leucocytes in semen.

There are difficulties associated with round cells from not only a laboratory perspective but also clinically. Differentiating round cells into leucocytes and germ cells requires expertise, and clinically, the impact on fertility and any subsequent actions have not been clearly defined for laboratory or clinician use.

This paper aims to review the possible importance of recognising round cells, differentiating between cell types and to address this from both a laboratory and clinical perspective.

## The Importance of Leucocytes in the Immune Response and its Relation to Semen

It is useful to understand the origins of blood cell lineage to comprehend the significance of leucocytes in blood and semen. Blood lines originate from haemocytoblasts, the multipotential haematopoietic stem cells residing in the bone marrow within central bone cavities (medulla) (5). These differentiate into one of two cell lines: myeloid or lymphoid progenitors. The myeloid cell line gives rise to erythrocytes, mast cells and the granule-containing cells (basophils, neutrophils, eosinophils, monocytes and macrophages) as well as megakaryocytes. The lymphoid cell line gives rise to the natural killer cells (NKC), T and B lymphocytes and plasma cells.

Leucocytes are normally present in semen and constitute 13% of non-spermatozoan cells, with differential proportions being neutrophils 12%, macrophages 0.9% and lymphocytes 0.1% (6).

During an infection, the human body mounts a defence that responds to the type and pathogenesis of the infective agent. The presence of >1 × 10^6^/ml leucocytes in semen is usually assumed to signify a genitourinary (GU) tract infection, accessory gland infection or inflammatory process (7, 8). The reported prevalence of bacteriospermia varies widely (6–68%) (9) but in approximately 50–80% of leucocytospermic samples, no microbial infection can be found (10). There are limitations with routine microbiological culture; standard operating procedures (for example UK Standards for Microbiology Investigations (11)) and associated interpretative guidelines are often limited and do not include uncommon pathogens and other infective agents as well as the normal rich seminal fluid microbiome. Multiplex PCR and next generation sequencing techniques have begun to yield more information (9, 12).

Where present, bacterial causes for prostatitis regularly include *Escherichia coli*, *Pseudomonas aeruginosa, Klebsiella*, *Enterococcus*, *Enterobacter*, *Proteus* and *Serratia* species (13). The most commonly found organisms in semen cultures appear to originate from skin flora contamination (14–16) with a proportion of symptomatic prostatitis patients testing positive for *Escherichia coli* (13). In male accessory gland (seminal vesicles, prostate and bulbourethral gland) infections (MAGI), there is often associated leucocytospermia, an increase in reactive oxygen species (ROS) and a decrease in sperm quality and function (17, 18). Clinically, the presence of infection in the accessory glands can lead to obstruction, orchitis and impairment of seminal fluid production (17). Ejaculatory duct obstruction (EDO) may be apparent when analysing a semen sample, as demonstrated by a combination of low seminal pH, low volume and azoospermia. In addition to *E.coli*, several organisms may contribute to MAGI, including gram-negative bacteria *Neisseria gonorrhoea*, *Chlamydia trachomatis*, *Ureaplasma urealyticum* and the fungal infection *Candida albicans* (17, 18).

In the event of a bacterial infection, the immune response may initiate any of three main modes of defence: complement pathway, phagocytic initiation and cell-mediated immunity (part of the innate immune response) (19). There is evidence that bacterial-induced UTIs rely heavily on initial neutrophil recruitment in response to epithelial cell chemokine production (20). Neutrophils are classed as peroxidase positive cells. Peroxidase positivity is limited to granulocytes that have intrinsic myeloperoxidase (MPO) (21). MPO is important as it oxidises substances including chloride ions (Cl^−^) and bromide ions (Br^−^) into reactive products including non-radicals such as hypochlorous acid (HOCL) as well as radicals (molecules that contain at least one unpaired electron) (21). Neutrophils are also able to use a nicotinamide adenine dinucleotide phosphate (NADPH) oxidase complex, Nox2, to give rise to oxidative bursts of superoxides (21). These systems are complex but aid in the destruction of invading microorganisms within phagosomes, change the behaviour of other cells in the vicinity and cause inflammation. These cells are not necessarily associated with an immune response to an initial viral infection.

Infections from viruses originate from the urethra or from the vascular system during an infection (22). Influenza, for example, has been investigated thoroughly for its impact on sperm parameters specifically due to the febrile aspect of the infection. There is evidence showing the presence of round cells in semen following a fever (23) and other evidence suggests that viral infections may result in leucocyte presence in human semen (24, 25). Viral infections can lead to several immune responses including class I major histocompatibility complex protein (MHC class I) initiation and the destruction by cytotoxic T cells; recruitment of natural killer cells (for when viruses are evading the MHC Class I complex pathway); interferon production; antibodies and phagocytosis (26). The evidence is limited in the appearance of leucocytes in semen following an infection external to the urothelial tract, but this should be considered, especially with the latter end-phase of viral infections being humoral antibody production from B lymphocytes.

In post-vasectomy samples, the number of white blood cells in semen decreases by 85–90%, possibly indicating that the vast majority of leucocytes originate from the testis and epididymis (22). A small population of leucocytes is considered normal and are probably involved in the elimination of degenerating cells (27), but it is suggested they are necessary for sperm function, as abnormally low numbers of leucocytes in the semen negatively affect fertilisation capacity in *in-vitro* fertilisation (28) and sperm capacitation is regulated by low levels of ROS produced by leucocytes in addition to sperm (29).

ROS remain an important aspect of male fertility research and discussions. ROS are highly reactive oxidising agents that potentially impact sperm quality and function (30). It is important to note that other sources of ROS in human ejaculate exist outside of leucocytes, including damaged sperm, immature sperm and germ cells (31). As well as being necessary for normal sperm function, they can result in oxidative stress (OS) when there is an imbalance in the redox system in humans. The impact of an over-production of ROS can cause lipid peroxidation, decrease in sperm motility, increase in DNA sperm fragmentation and sperm apoptosis (32).

## The Presence of Sperm Cell Progenitors in Semen

The other subset of “round cells” that must be considered is germ cells, which are present from spermatogenesis (the process whereby immature germ cells, spermatogonia, differentiate into spermatozoa) (33). The process of spermatogenesis involves mitosis, meiosis, spermiogenesis, spermiation and release, and has been extensively covered in andrology literature. Germ cells have been stated as being the predominant cell types when round cells are detected, and have been reported as constituting 84% of non-spermatozoan cells, (respectively 43% anucleate bodies, 22.2% spermatids, 18.8% spermatocytes plus anucleate bodies with organelles) (6), although no normal levels have officially been defined for reference (24). It has been suggested that the emergence of round cells, particularly germ cells, may be the result of failed spermiogenic completion or active regeneration of the epithelium after transient attacks such as that from influenza (34). There may also be an increase in germ cell shedding with testicular maturation arrest. Here, during early arrest, germ cells do not progress past the primary spermatocyte level and in late arrest, germ cells do not progress past the round spermatid level (35). A depletion in the supply of mature sperm along with a higher relative percentage of immature sperm and germ cells has also been noted in men with prolonged frequent ejaculations (36), which can be due to severe masturbation compulsion (37) or misinformation regarding intercourse frequency when trying to conceive. This is important in understanding the reasons for the patient’s cause of infertility and can be confirmed with adequate review of the patient.

## Significance and Clinical Importance of Leucocytospermia in Fertility and Health Actions to Consider

Identification of cells in the semen sample can lead to an understanding of either current or passing infections along with information regarding spermatogenesis and lifestyle factors which may adversely affect fertility. Ideally, round cell identification should lead to differentiation of germ cells from leucocytes, followed by review of the patient. All semen samples should be accompanied by a request form and ideally some key questions answered regarding the patient’s health relevant for the test (see Table 1 for considerations). Having a comprehensive patient discussion prior to the semen analysis will supplement any findings noted from laboratory tests.

**TABLE 1:** Key minimal health questions to consider for semen analysis with fertility investigations.

Question	Justification
Have you had any illnesses within the past 6 months?	To ascertain if any febrile illnesses or other infections that may contribute to sperm quality and findings (38).
How often do you ejaculate through either sexual intercourse or masturbation?	Discussed within text.
Have you had any surgery?	Some abdominal surgery may cause disruption or the reproductive tract. At the very least, the surgery may have been due to a GU issue.
Have you ever had any cancer treatment?	Cancer treatment may include the use of cytotoxic drugs. These can induce severe oligozoospermia, azoospermia or an increase in germ cell populations (35).
Do you take any recreational drugs, smoke or drink alcohol?	Several studies document the effects of recreational drugs and alcohol on sperm parameters and round cells (39–42). The use of these drugs may also impact sexual and endocrine function.
Have you been diagnosed with any conditions? This may be genetic or otherwise.	This is an open question but can be tailored and adapted depending on the service. It is aimed at understanding if there are any issues that may impact fertility or findings in semen.

It is vital that genital tract infections are diagnosed using a combination of accurate history taking alongside symptoms (this may be complemented by physical examination) (43). Symptoms may include changes in the voiding process, urethral discharge, pain (particularly at ejaculation) and testicular/local tenderness. If a patient is asymptomatic, the identification of infection will be much more difficult, and may not necessarily be warranted unless there is suggestive evidence to demonstrate potential exposure. One of the primary areas of concern for patients where high numbers of leucocytes are detected in their semen, is the possible detrimental impact they have either on the direct functioning of sperm from a microscopic perspective, as well as the physiology of the patient which may lead to obstructions in the passage of sperm (due to inflammatory processes) or/and by impeding spermatogenesis.

Symptoms of bacterial prostatitis include dysuria, varying frequency of urination and increased urine retention and can result in more severe manifestations such as pain, fevers, chills, malaise and nausea (13). It is estimated that the majority of cases are acquired, although adequate history review may suggest iatrogenic causes such as catheterisation or transrectal biopsy procedures (13).

Non-gonococcal urethritis (NGU) can be caused by a number of infections (44) (see Table 2). Patients may present with urethral discharge, dysuria, urethral stinging/itching but many may be asymptomatic (45). The presence of discharge results in purulent semen containing massed polymorphonuclear leucocytes (neutrophils), easily demonstrated using direct spread and rapid staining techniques, or detecting >1+ on a mid-stream urine dipstick test (46). Men with long-term or recurrent NGU may develop epididymo-orchitis (EO) as a complication. EO can directly damage the testis and subsequent spermatogenesis, as well as impacting the testicular endocrine function (47, 48).

**TABLE 2:** Causes of non-gonococcal urethritis (NGU) in men.

Aetiology of NGU	Proportion of cases (%)
*Chlamydia trachomatis*	20–50%
*Mycoplasma genitalium*	10–30%
*Ureaplasma urealyticum*	5–10%
*Trichomonas vaginalis* (UK)	1–5%
Urinary tract infection	1–5%
Adenovirus	2–4%
Herpes simplex virus	2–3%
Miscellaneous	Less than 5%

In studies of infertile couples, several lifestyle factors have been shown to increase the seminal leucocyte population without obvious evidence of past or current infection. These include cigarette smoking, use of marijuana and heavy alcohol drinking (49). A study of 65 men from a fertility clinic in USA found a 48% increase in leucocytes in infertile men who smoked (50), a larger Austrian study of 1,104 men confirmed a significantly higher percentage of ejaculates (23.5%) with >1 × 10^6^/ml peroxidase positive round cells in smokers compared with non- or ex-smokers (51), and a later study of 112 infertile men in Brazil showed levels of leucocytospermia increased as cigarette smoking increased (52).

**FIGURE 1:**
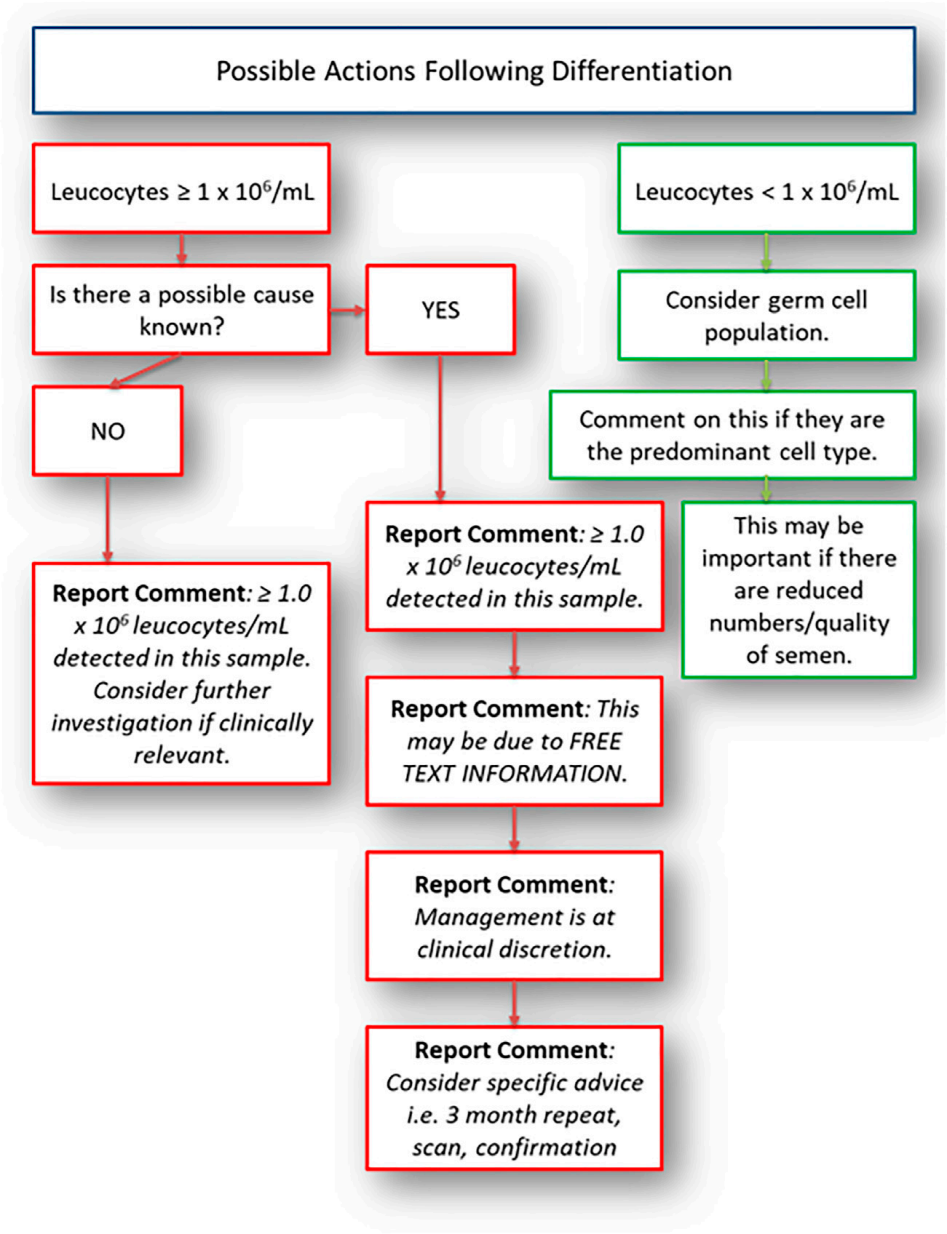
Possible actions following round cell differentiation. This should be used as a guide only.

There should also be considerations for other causes of leucocytes and germ cells; these are given in Table 3. In any case, laboratory identification of round cells should lead to differentiation followed by review of the patient. This is best undertaken by the referring clinician unless the healthcare professional or scientist has had further training to do this and has the permission of the clinician responsible for that patient. The possible outcome from initial detection of “round cells” needs to be in line with the patient symptoms, history, semen quality and the management possibilities of the couple. Differentiation should take place if significant numbers of round cells are seen (estimation of numbers using a haemocytometer is a valid method). The concentration considered significant should be determined based on guidelines available and the needs of the service users; the authors consider a concentration of ≥1.0 × 10^6^ round cells/ml to be significant, in line with WHO 2021 (2). The laboratory actions at this stage will depend on the patient history, previous semen analysis and outcome of differentiation. A suggested pathway is given in Figure 1. This pathway will be determined by training, competency, clinical supervision and limitations of the methods/staffing. The pathway in Figure 1 is suggested in the absence of laboratory specific direction, to support reporting and actions relating to the detection of round cells. Very few related guidelines exist; and where they do, such as the European Association of Urology (EAU) (53) and WHO 2021 (2), they suggest clinical considerations and several factors that can impact the ejaculate. These include medication, endocrine status, anatomy (testicular size), illnesses and non-prescribed drugs. They fail to fully provide advice outside of positive peroxidase testing and subsequent microbiological cultures by which a diagnostic laboratory can follow and develop. The laboratory therefore can supplement the information known by the referring clinician. Comments and advice should be added to the user handbook or appropriate method used for communicating with users regarding information and interpretation. If a clinician reviews the patient (including a physical examination) and feels that there are symptoms that require further investigation, they should consider testing locally or referring the patient to a GU clinic for specialist testing, partner tracing and treatment if required before proceeding with a repeat semen analysis or assisted reproductive technology (ART) referral. In patients where there are no identifiable causes, the clinician may consider antioxidants, lifestyle changes and a repeat after considerable changes have been established. On occasions, it may be necessary to undertake radiological diagnosis, although the type required will need to be relevant and the risks/benefits of any radiation intervention should be considered by the person responsible. Ultrasound scans are often a useful tool in diagnostics but is not indicated for all patients.

**TABLE 3:** Causes of leucocytes and germ cells in semen.

Leucocytes in semen	Germ cells in semen
Bacterial infection	Varicocele
Viral infection	Cytotoxic treatment i.e., chemotherapy
Recovery from illness	Cryptorchidism
Stress and acute illness	Maturation arrest
Chronic illness	Prolonged frequent ejaculation
Recreational drug use including alcohol and smoking	
Trauma	

## Laboratory Methods for Identifying Round Cells and Images

Analysis of round cells will often be undertaken on stained slides during morphological examination. In some instances, scientists will review the concentration of round cells using the same method applied for concentration analysis (gold standard being stated as the improved Neubauer haemocytometer) (2). At this point, the decision will be to decide whether further identification and differentiation is required or whether the round cell estimation will be used. Many laboratories will not give any clinically relevant information in the final report, but if calculated to be ≥1 × 10^6^/ml may simply include a comment stating that round cells are present. This gives no indication of the proportion of leucocytes or germ cells in the reporting of round cells. The issues arise from the specific methods on how laboratories will differentiate if they want to, and if they do, what does the end-user do with this information?

**FIGURE 2:**
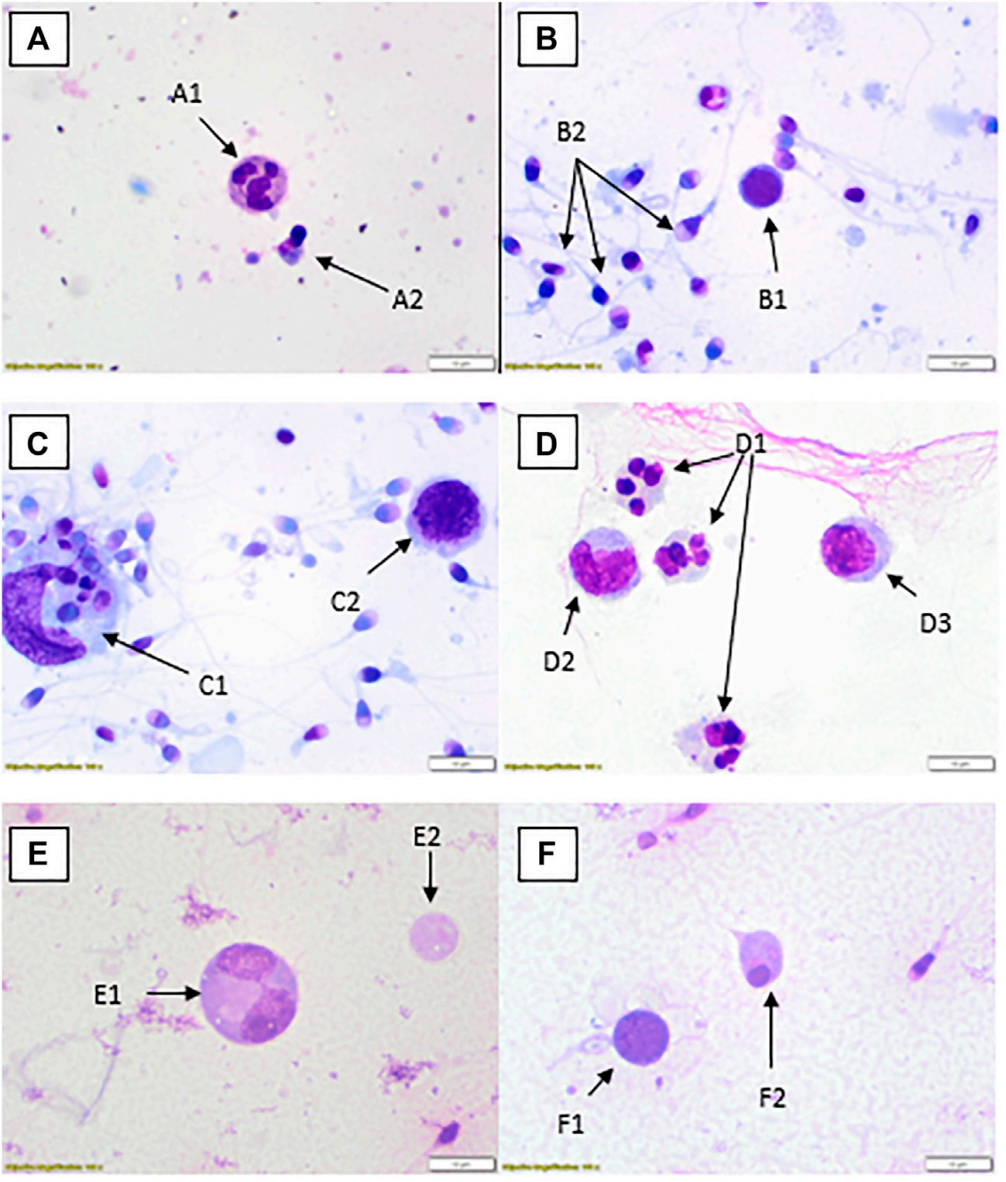
Rapid Romanowsky stained preparations with ×100 objective and ×10 optics. **(A)** Neutrophil (A1), spermatid (A2). **(B)** Possible lymphocyte (B1), multiple spermatozoa (B2). **(C)** Large phagocytic macrophage with engulfed material (C1), lymphocyte (C2). **(D)** Three neutrophils (D1), two monocytes (D2, D3). **(E)** Dividing spermatocyte (E1), cytoplasmic remnant (E2). **(F)** Possible lymphocyte (F1), spermatid (F2).

**FIGURE 3:**
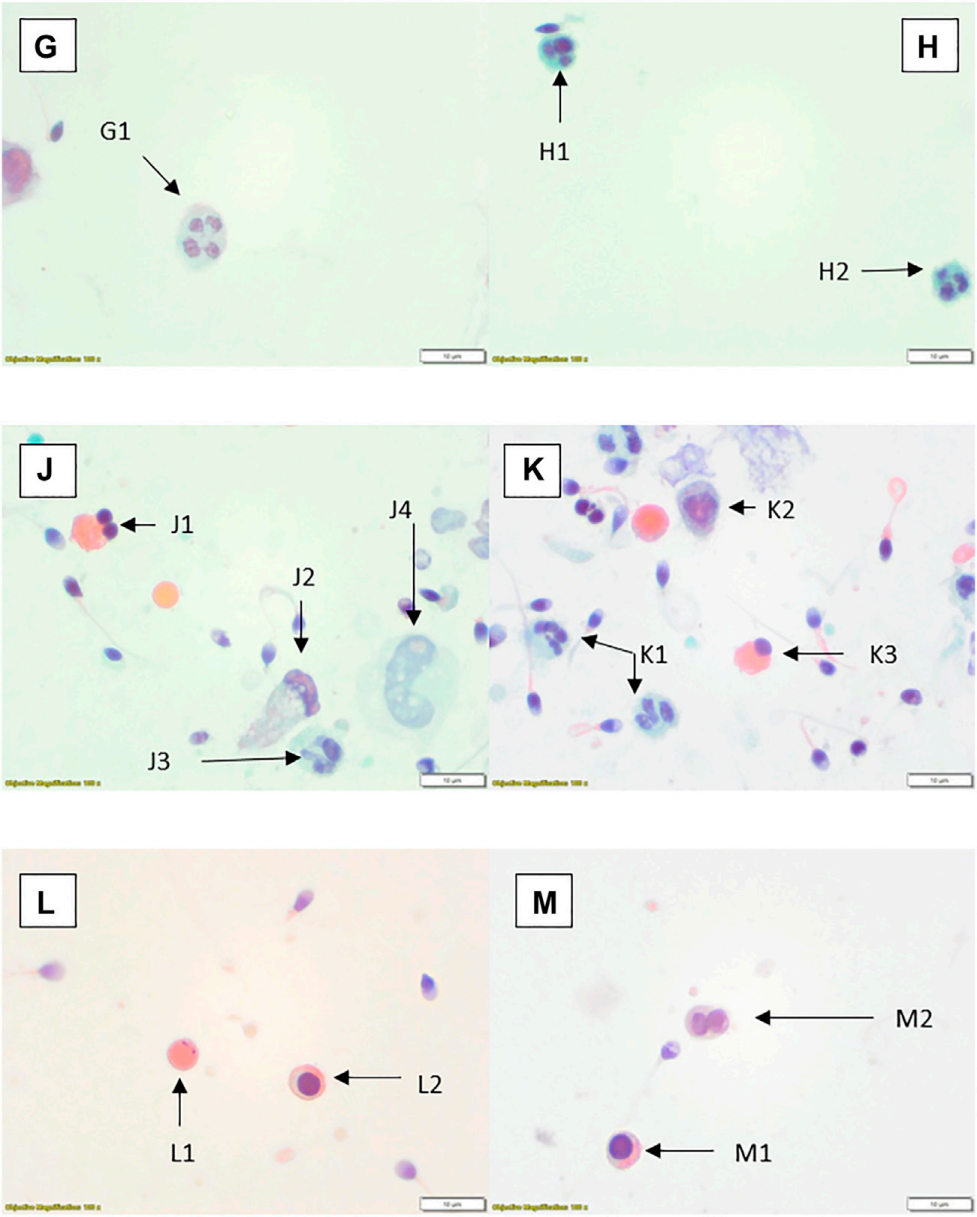
Papanicolaou stained preparations with ×100 objective and ×10 optics. **(G)** Neutrophil (G1). **(H)** Two small neutrophils (H1, H2). **(J)** Spermatid (J1), possible degenerate/damaged neutrophil (J2), neutrophil (J3), possible degenerate monocyte (J4). **(K)** Neutrophils (K1, K2), spermatid (K3). **(L)** Cytoplasmic remnant (L1), spermatocyte (L2). **(M)** Spermatocyte (M1), spermatids (M2).

**FIGURE 4:**
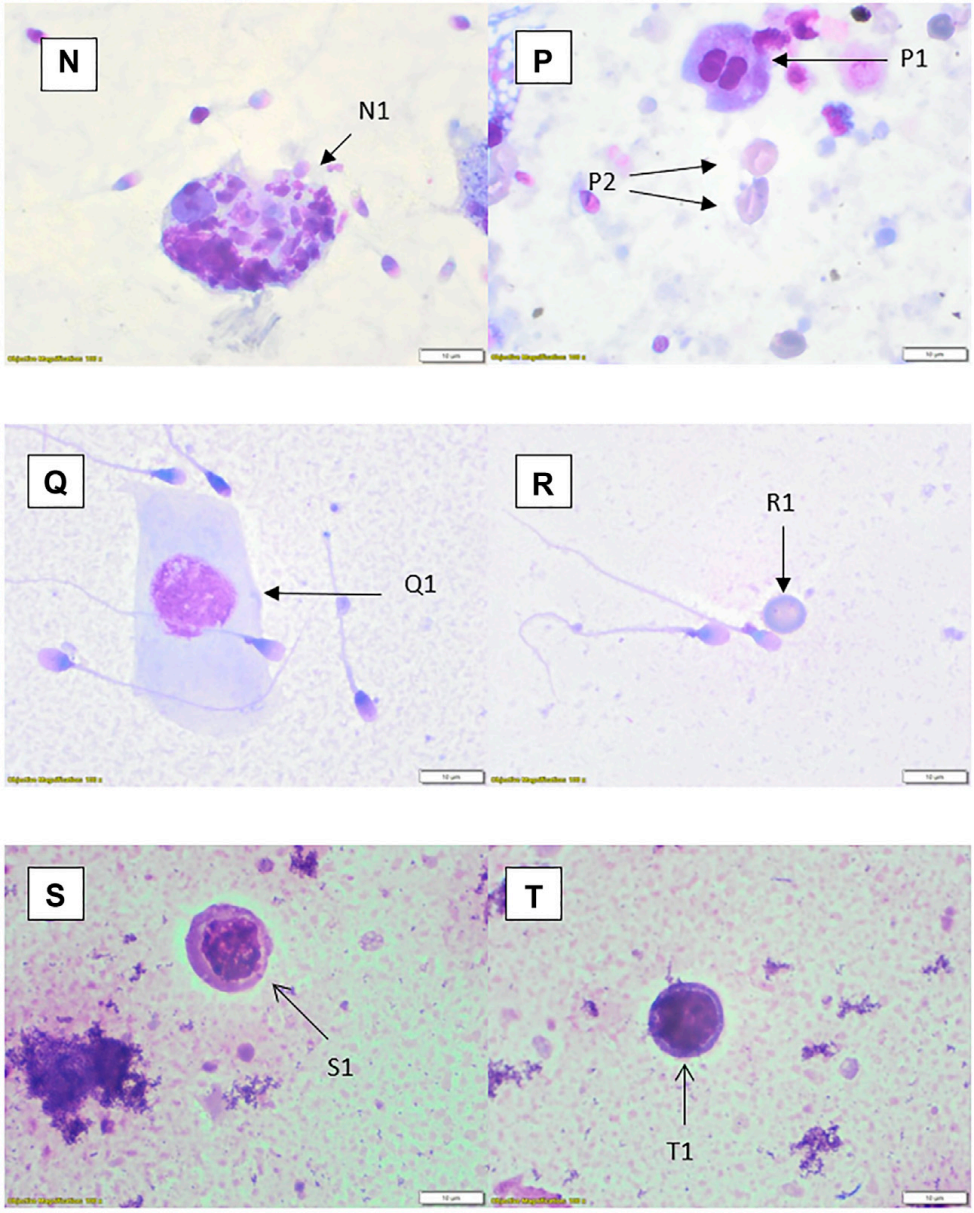
Rapid Romanowsky stained preparations with ×100 objective and ×10 optics. **(N)** Phagocytic macrophage (N1). **(P)** Spermatid (P1), erythrocytes (P2); centrifuged sample. **(Q)** Squamous epithelial cell (Q1). **(R)** Erythrocyte (R1). **(S)** Small monocyte (S1). **(T)** Possible lymphocyte (T1).

**FIGURE 5:**
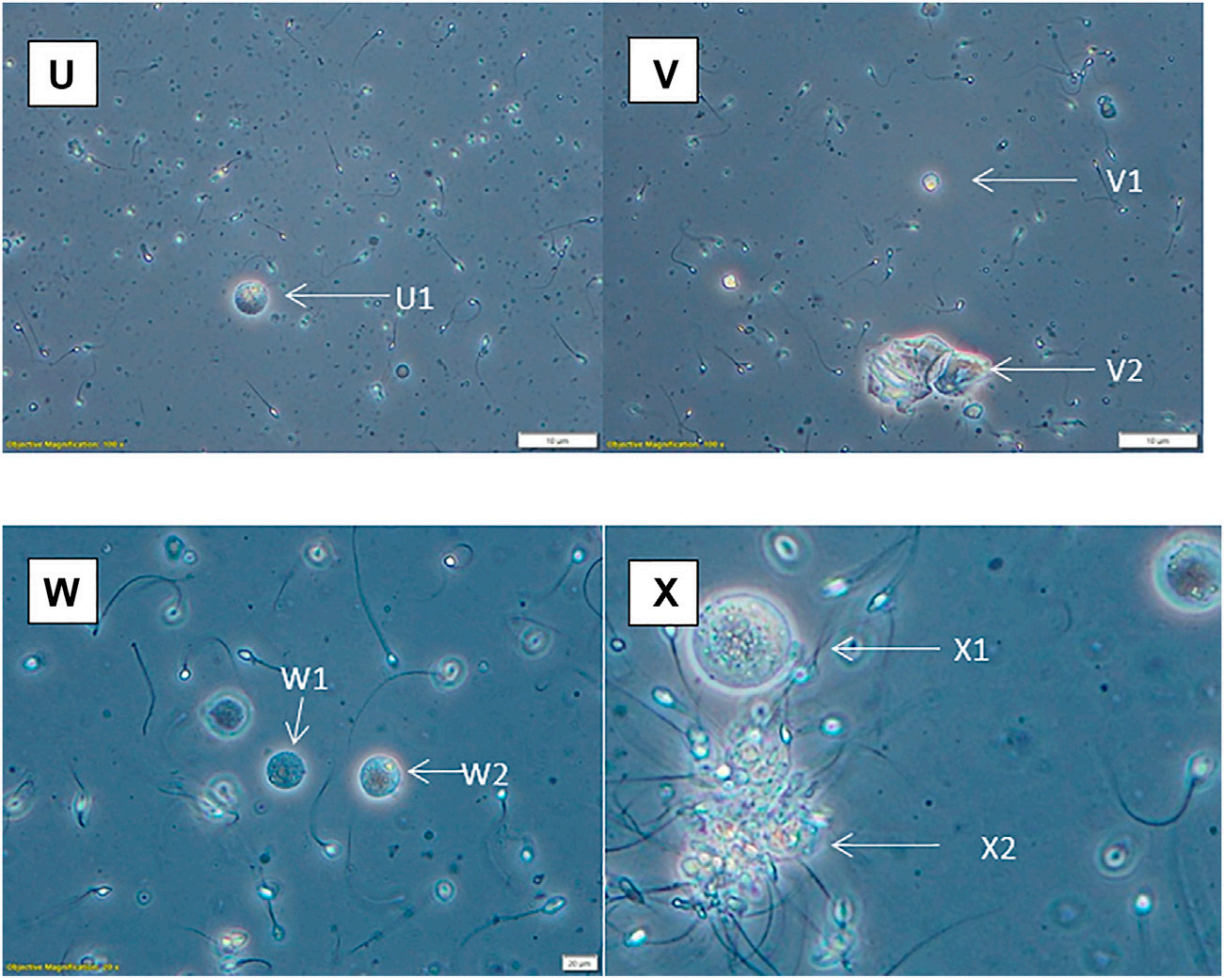
Phase contrast with ×20 optics (except X, which has been artificially enlarged by 50%). **(U)** Possible spermatid (U1). **(V)** Spermatid (V1), squamous epithelial cells (V2). **(W)** Spermatids (W1, W2). **(X)** Spermatocyte (X1), aggregated sperm with debris (X2).

**FIGURE 6:**
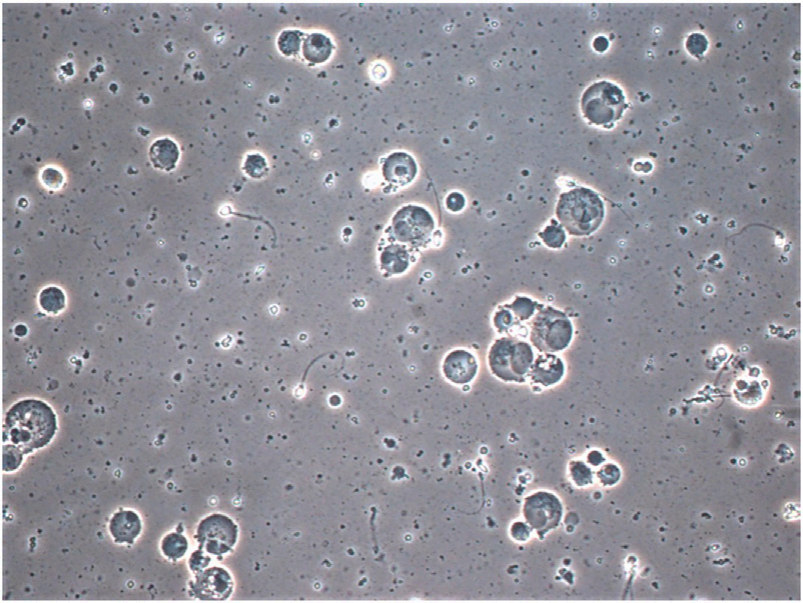
Photomicrograph of semen from patient with prolonged ×10 daily ejaculation (phase contrast × 40 objective with × 20 optics) showing multiple spermatocytes and spermatids and depleted sperm. The much more rounded and uniform appearance of the nuclear structures, which are separate are clearly distinguished from neutrophils which have coarsely clumped chromatin lobes joined by thin chromatin bridges as seen for example in Figure 2, cells A1 and D1. Cells containing spermatid nuclei may also varying stages of spermatid to sperm maturation and vacuoles due to sperm emergence.

WHO suggested methods for quantification of round cells may not be practical or suitable for the laboratory. There are many methods that can be applied for the assessment of leucocytes including seminal granulocyte elastase test, immunochemistry and the peroxidase test (54). Appropriate validation and verification of any method must be considered, particularly if accreditation to standards for this aspect is to be included. The method for validation/verification will depend on the test to be used, which would include morphological differentiation. The next stage in the process, should a laboratory choose to do so, is to differentiate these cells into leucocytes and germ cells. The simplest recommended method is to stain the peroxidase enzyme found in granulocytes. The limitation with staining using the peroxidase enzyme is that it will only identify an active disease or infection rather than one in its later stages such as a patient with chronic infection (55). There are many viable options for leucocyte assessment given in WHO 2021 or other scientific papers (54). The often less-favourable option is the use of the stained preparations and morphological distinction. Although subjective, there is no reason why the method cannot be applied well and give clinically relevant information, particularly when other methods such as the peroxidase test will miss non-granule containing cells or those where they have already released their active compounds. As discussed earlier, cell types can be important in determining the potential cause of infection or support direction of investigations as appropriate. This is not to say peroxidase testing is not valuable, but limitations of any test should be considered. Staining and differentiation can lead to misinterpretation of cells, particularly as the preparation differs to routine blood smears which can be used in the determination of white blood counts. These pitfalls in the cytological subjective interpretation of cells, has been a discussion point among cytology disciplines, in particular cervical cytology (56), for many years. In stained semen spreads, for example, there can be difficulties in visually identifying small chromatin bridges between neutrophil nuclei segments when comparing them to multinucleated spermatids (57). This is where peroxidase assessments could support subjective interpretation comparatively to staining methods. It is also worth noting that WHO 2021 guidelines state that it is “not possible to distinguish between leukocytes and immature germ cells with a high degree of certainty (2)” and they recommend an alternative method of analysis. It is worth noting that the reference for this decision is from 1966 (58) and possibly more information and study is required. A small study from 2003 (59) reviewed the possibility of peroxidase positivity and elastase levels (these proteases rise with increased granulocyte levels as part of the inflammation process) and found no correlation between the two tests, indicating that it may not necessarily be a good determinant of leucocytes in semen. Staining procedures are generally lower-cost and can be used with training to determine overall cell populations, although caution should always be taken in the confidence of the values for any method including stained slide interpretation. Alternative methods are not discussed in this paper but should be considered by a laboratory in their review of appropriate laboratory procedures. The photomicrographical images included (Figures 2–6) will give laboratory staff a small atlas for the identification of different cells using numerous methods. The image set is not comprehensive but offers guidance for laboratories.

## Staining Protocols for Photomicrographs

Rapid staining procedure was undertaken using Cell Path Limited Rapid Romanowsky Stain kit (SKU: RHS-705-3X1) following full air-drying of semen spreads. The method included a minimum fixation step of 30 s in Solution 1 (alcohol fixative) followed by two 5 s complete submersions in Solution 2 (Eosin Y) and Solution 3 (Azure II and sodium azide). Two 5 s water washes were applied after staining and slides were left to dry naturally before examination under oil-immersion bright-field microscopy. Papanicolaou staining procedures were based on pre-existing cytopathology methods i.e., a modified Papanicolaou staining protocol deviating from WHO 2021 methods (2). This is available upon request but involves sequential staining steps involving industrial methylated alcohol, haematoxylin, water, acid water, lithium carbonate, OG6 stain, EA50 and xylene prior to mounting. The staining components were provided by Cell Path Limited.

## Discussion

The importance and significance of leucocytes in semen has been discussed in numerous studies. Whilst this is well-known, laboratories find it difficult to ensure that they are giving users adequate information for interpretation when reports are purely numerical. This increases the risk of either inappropriate treatment, including unnecessary prescribing of antibiotics, or for missing opportunities to improve men’s health, sperm quality and the pathway for every person involved in trying to conceive. Appropriate review of the patient should be considered alongside laboratory findings to direct clinicians’ use of the correct diagnostic tools. The importance of providing a sensitive approach to reporting and patient review is paramount, as patients may misunderstand the relevance, especially with regard to GU infections, which may not be sexually transmitted. Laboratories can support users by providing them with reliable results, giving adequate advice where needed and ensuring reports are clear. Where clarity or advice is required, especially where many unusual or abnormal-looking cells are noted, haematologists and cytopathologists can be utilised to support andrologists in the identification process, or the use of consensus opinion may be an approach worth taking. Training and competency assessments, along with validation if required, will always be important to provide assurance of a scientist’s ability to recognise cells. The overall aim should be to give the user an idea of the majority cell population as opposed to categorically differentiating into individual white blood cell categories and germ cell types. In laboratories where highly trained staff participate in semen analysis, they can undertake this task with fewer additional extended tests. Taking this alongside current WHO 2021 guidelines which direct laboratories to clinical decision values as opposed to just the prescriptive lower reference limits and fifth centiles, it will ensure that direction for management is at the core of analysis. This will ensure appropriate clinical review with the patient so that the correct management plans can be agreed and undertaken.

## References

[1] WHO. Laboratory Manual for the Examination of Human Semen and Sperm-Cervical Mucus Interaction. 1st Edn. Singapore: Press Concern (1980). p. 43.

[2] WHO. WHO Laboratory Manual for the Examination and Processing of Human Semen. 6th Edn. Geneva: World Health Organisation (2021).

[3] Ravitsky V Kimmins S. The Forgotten Men: Rising Rates of Male Infertility Urgently Require New Approaches for its Prevention, Diagnosis and Treatment. Biol Reprod (2019) 101(5):872–4. 10.1093/biolre/ioz161 31553040 PMC6877781

[4] Goldman AS Prabhakar BS. Immunology Overview. In: Baron S, editor. Medical Microbiology. Galveston (TX): University of Texas Medical Branch at Galveston (1996). 21413267

[5] Birbrair A Frenette PS. Niche Heterogeneity in the Bone Marrow. Ann N.Y Acad Sci (2016) 1370(1):82–96. 10.1111/nyas.13016 27015419 PMC4938003

[6] Smith DC Barratt CL Williams MA. The Characterisation of Non-sperm Cells in the Ejaculates of fertile Men Using Transmission Electron Microscopy. Andrologia (1989) 21(4):319–33. 2782636

[7] Sandoval JS Raburn D Muasher S. Leukocytospermia: Overview of Diagnosis, Implications, and Management of a Controversial Finding. Middle East Fertil Soc J (2013) 18(3):129–34. 10.1016/j.mefs.2013.02.004

[8] Brunner RJ Demeter JH Sindhwani P. Review of Guidelines for the Evaluation and Treatment of Leukocytospermia in Male Infertility. World J Mens Health (2019) 37(2):128–37. 10.5534/wjmh.180078 30644236 PMC6479086

[9] Farahani L Tharakan T Yap T Ramsay JW Jayasena CN Minhas S. The Semen Microbiome and its Impact on Sperm Function and Male Fertility: A Systematic Review and Meta‐Analysis. Andrology (2021) 9(1):115–44. 10.1111/andr.12886 32794312

[10] Gambera L Serafini F Morgante G Focarelli R De Leo V Piomboni P. Sperm Quality and Pregnancy Rate after COX-2 Inhibitor Therapy of Infertile Males with Abacterial Leukocytospermia. Hum Reprod (2007) 22(4):1047–51. 10.1093/humrep/del490 17208944

[11] NHS. Investigation of Genital Tract and Associated Specimens*.* UK Standards for Microbiology Investigations, Standards Unit, Microbiology Services. London: Public Health England (2017). p. 1–40.

[12] Ventimiglia E Capogrosso P Boeri L Cazzaniga W Matloob R Pozzi E Leukocytospermia Is Not an Informative Predictor of Positive Semen Culture in Infertile Men: Results from a Validation Study of Available Guidelines. Hum Reprod Open (2020) 2020(3):hoaa039. 10.1093/hropen/hoaa039 32995564 PMC7508024

[13] Coker TJ Dierfeldt DM. Acute Bacterial Prostatitis: Diagnosis and Management. Am Fam Physician (2016) 93(2):114–20. 26926407

[14] Cottell E Harrison RF McCaffrey M Walsh T Mallon E Barry-Kinsella C. Are Seminal Fluid Microorganisms of Significance or Merely Contaminants? Fertil Steril (2000) 74(3):465–70. 10.1016/s0015-0282(00)00709-3 10973639

[15] Cottell E Lennon B McMorrow J Barry-Ḱinsella C Harrison RF. Processing of Semen in an Antibiotic-Rich Culture Medium to Minimize Microbial Presence during *In Vitro* Fertilization. Fertil Steril (1997) 67(1):98–103. 10.1016/s0015-0282(97)81863-8 8986691

[16] Stovall DW Bailey LE Talbert LM. The Role of Aerobic and Anaerobic Semen Cultures in Asymptomatic Couples Undergoing *In Vitro* Fertilization: Effects on Fertilization and Pregnancy Rates. Fertil Steril (1993) 59(1):197–201. 10.1016/s0015-0282(16)55639-8 8419208

[17] Krause W. Male Accessory Gland Infection. Andrologia (2008) 40(2):113–6. 10.1111/j.1439-0272.2007.00822.x 18336461

[18] La Vignera S Vicari E Condorelli RA D’Agata R Calogero AE. Male Accessory Gland Infection and Sperm Parameters (Review). Int J Androl (2011) 34(5pt2):e330–e347. 10.1111/j.1365-2605.2011.01200.x 21696400

[19] Bellanti JA. Cell-Mediated Immunity. J Allergy Clin Immunol (1989) 84(6 Pt 2):1036–9. 10.1016/0091-6749(89)90147-4 2600335

[20] Haraoka M Hang L Frendéus B Godaly G Burdick M Strieter R Neutrophil Recruitment and Resistance to Urinary Tract Infection. J Infect Dis (1999) 180(4):1220–9. 10.1086/315006 10479151

[21] Winterbourn CC Kettle AJ Hampton MB. Reactive Oxygen Species and Neutrophil Function. Annu Rev Biochem (2016) 85(1):765–92. 10.1146/annurev-biochem-060815-014442 27050287

[22] Keck C Gerber-Schäfer C Clad A Wilhelm C Breckwoldt M. Seminal Tract Infections: Impact on Male Fertility and Treatment Options. Hum Reprod Update (1998) 4(6):891–903. 10.1093/humupd/4.6.891 10098479

[23] Sergerie M Mieusset R Croute F Daudin M Bujan L. High Risk of Temporary Alteration of Semen Parameters after Recent Acute Febrile Illness. Fertil Steril (2007) 88(4):970–7. 10.1016/j.fertnstert.2006.12.045 17434502

[24] Fedder J. Nonsperm Cells in Human Semen: With Special Reference to Seminal Leukocytes and Their Possible Influence on Fertility. Arch Androl (1996) 36(1):41–65. 10.3109/01485019608987883 8824667

[25] Li H Xiao X Zhang J Zafar MI Wu C Long Y Impaired Spermatogenesis in COVID-19 Patients. EClinicalMedicine (2020) 28:100604. 10.1016/j.eclinm.2020.100604 33134901 PMC7584442

[26] Koyama S Ishii KJ Coban C Akira S. Innate Immune Response to Viral Infection. Cytokine (2008) 43(3):336–41. 10.1016/j.cyto.2008.07.009 18694646

[27] Cavarelli M Le Grand R. The Importance of Semen Leukocytes in HIV-1 Transmission and the Development of Prevention Strategies. Hum Vaccin Immunother (2020) 16(9):2018–32. 10.1080/21645515.2020.1765622 32614649 PMC7553688

[28] Tomlinson MJ Barratt CLR Cooke ID. Prospective Study of Leukocytes and Leukocyte Subpopulations in Semen Suggests They Are Not a Cause of Male Infertility. Fertil Steril (1993) 60(6):1069–75. 10.1016/s0015-0282(16)56412-7 8243688

[29] Ford WC. Male Infertility: Tales of Progress and Frustration. Hum Fertil (2002) 5(1 Suppl. l):S53–60. 10.1080/1464727022000199931 11897917

[30] Agarwal A Sharma R Nallella K Thomasjr A Alvarez J Sikka S. Reactive Oxygen Species as an Independent Marker of Male Factor Infertility. Fertil Steril (2006) 86(4):878–85. 10.1016/j.fertnstert.2006.02.111 17027357

[31] Gil-Guzman E Ollero M Lopez MC Sharma RK Alvarez JG Thomas AJ Differential Production of Reactive Oxygen Species by Subsets of Human Spermatozoa at Different Stages of Maturation. Hum Reprod (2001) 16(9):1922–30. 10.1093/humrep/16.9.1922 11527899

[32] Henkel R Kierspel E Stalf T Mehnert C Menkveld R Tinneberg H-R Effect of Reactive Oxygen Species Produced by Spermatozoa and Leukocytes on Sperm Functions in Non-leukocytospermic Patients. Fertil Steril (2005) 83(3):635–42. 10.1016/j.fertnstert.2004.11.022 15749492

[33] Neto FTL Bach PV Najari BB Li PS Goldstein M. Spermatogenesis in Humans and its Affecting Factors. Semin Cell Develop Biol (2016) 59:10–26. 10.1016/j.semcdb.2016.04.009 27143445

[34] Palermo GD Neri QV Cozzubbo T Cheung S Pereira N Rosenwaks Z. Shedding Light on the Nature of Seminal Round Cells. PLoS One (2016) 11(3):e0151640. 10.1371/journal.pone.0151640 26982590 PMC4794220

[35] Halder A Kumar P Jain M Iyer VK. Copy Number Variations in Testicular Maturation Arrest. Andrology (2017) 5(3):460–72. 10.1111/andr.12330 28217965

[36] Mayorga-Torres BJM Camargo M Agarwal A du Plessis SS Cadavid ÁP Cardona Maya WD. Influence of Ejaculation Frequency on Seminal Parameters. Reprod Biol Endocrinol (2015) 13(1):47. 10.1186/s12958-015-0045-9 25994017 PMC4445565

[37] Cui Z Mo M Chen Q Wang X Yang H Zhou N Pornography Use Could Lead to Addiction and Was Associated with Reproductive Hormone Levels and Semen Quality: A Report from the MARHCS Study in China. Front Endocrinol (2021) 12(1101):736384. 10.3389/fendo.2021.736384 PMC8461095 34566897

[38] Long S Dawe S Woodward B. Reduced Sperm Concentration in a Patient From a Suspected Post-Operative Infection: A Case Study British J Biomed Sci (2020) 77(3):148–51. 10.1080/09674845.2020.1732638 32091306

[39] Kulkarni M Hayden C Kayes O. Recreational drugs and male fertility Trends Urol Men’s Health (2014) 5(5):19–23. 10.1002/tre.414

[40] du Plessis SS Agarwal A Syriac A. Marijuana, phytocannabinoids, the endocannabinoid system, and male fertility J Assisted Reproduction Genet (2015) 32(11):1575–88. 10.1007/s10815-015-0553-8 PMC4651943 26277482

[41] Hsiao P Clavijo RI. Adverse Effects of Cannabis on Male Reproduction European Urol Focus (2018) 4(3):324–8. 10.1016/j.euf.2018.08.006 30146239

[42] Ajayi AF Akhigbe RE. The physiology of male reproduction: Impact of drugs and their abuse on male fertility Andrologia (2020) 52(9):e13672. 10.1111/and.13672 32542870

[43] Moi H Blee K Horner PJ. Management of Non-gonococcal Urethritis. BMC Infect Dis (2015) 15(1):294. 10.1186/s12879-015-1043-4 26220178 PMC4518518

[44] Shahmanesh M Moi H Lassau F Janier M. 2009 European Guideline on the Management of Male Non-gonococcal Urethritis. Int J STD AIDS (20092009) 20(7):458–64. 10.1258/ijsa.2009.009143 19541886

[45] Horner P Blee K O’Mahony C Muir P Evans C Radcliffe K. 2015 UK National Guideline on the Management of Non-gonococcal Urethritis. Int J STD AIDS (2016) 27(2):85–96. 10.1177/0956462415586675 26002319

[46] Fijak M Pilatz A Hedger MP Nicolas N Bhushan S Michel V Infectious, Inflammatory and ‘autoimmune’ Male Factor Infertility: How Do Rodent Models Inform Clinical Practice? Hum Reprod Update (2018) 24(4):416–41. 10.1093/humupd/dmy009 29648649 PMC6016649

[47] Schuppe H-C Pilatz A Hossain H Meinhardt A Bergmann M Haidl G Orchitis und Infertilität. Urologe (2010) 49(5):629–35. 10.1007/s00120-010-2256-1 20449780

[48] Bar-Chama N Fisch H. Infection and Pyospermia in Male Infertility. World J Urol (1993) 11(2):76–81. 10.1007/BF00182033 8343798

[49] Close CE Roberts PL Berger RE. Cigarettes, Alcohol and Marijuana are Related to Pyospermia in Infertile Men. J Urol (1990) 144(4):900–3. 10.1016/s0022-5347(17)39618-0 2398564

[50] Saleh RA Agarwal A Sharma RK Nelson DR Thomas AJ. Effect of Cigarette Smoking on Levels of Seminal Oxidative Stress in Infertile Men: A Prospective Study. Fertil Steril (2002) 78(3):491–9. 10.1016/s0015-0282(02)03294-6 12215323

[51] Trummer H Habermann H Haas J Pummer K. The Impact of Cigarette Smoking on Human Semen Parameters and Hormones. Hum Reprod (2002) 17(6):1554–9. 10.1093/humrep/17.6.1554 12042277

[52] Pasqualotto FF Umezu FM Salvador M Borges E Sobreiro BP Pasqualotto EB. Effect of Cigarette Smoking on Antioxidant Levels and Presence of Leukocytospermia in Infertile Men: a Prospective Study. Fertil Steril (2008) 90(2):278–83. 10.1016/j.fertnstert.2008.02.123 18462724

[53] Salonia A Bettocchi C Carvalho J Corona G Jones TH Kadioǧlu A European Association of Urology Sexual and Reproductive Health Guidelines. Eur Urol (2021) 80:333–57. 10.1016/j.eururo.2021.06.007 34183196

[54] Sharma R Gupta S Agarwal A Henkel R Fineli R Parekh N Relevance of Leukocytospermia and Semen Culture and its True Place in Diagnosing and Treating Male Infertility. World J Mens Health (2021) 39. 10.5534/wjmh.210063 PMC8987138 34169683

[55] Khan A Alsahli M Rahmani A. Myeloperoxidase as an Active Disease Biomarker: Recent Biochemical and Pathological Perspectives. Med Sci (2018) 6(2):33. 10.3390/medsci6020033 PMC6024665 29669993

[56] Torous VF Pitman MB. Interpretation Pitfalls and Malignant Mimics in Cervical Cytology. J Am Soc Cytopathol. (2021) 10(2):115–27. 10.1016/j.jasc.2020.06.005 32732114

[57] Johanisson E Campana A Luthi R Agostini AD. Evaluation of ‘Round Cells’ in Semen Analysis: A Comparative Study. Hum Reprod Update (2000) 6(4):404–12. 10.1093/humupd/6.4.404 10972527

[58] Freund M. Standards for the Rating of Human Sperm Morphology. A Cooperative Study. Int J Fertil (1966) 11:97–180. 5934263

[59] Sánchez R Villegas J Peña P Miska W Schill WB. Determination of Peroxidase Positive Cells in Semen: Is it a Secure Parameter for the Diagnosis of Silent Genital Infections? Revista Med de Chile (2003) 131(6):613–16. 12942588

